# Melatonin protects the heart and pancreas by improving glucose homeostasis, oxidative stress, inflammation and apoptosis in T2DM-induced rats

**DOI:** 10.1016/j.heliyon.2021.e06474

**Published:** 2021-03-12

**Authors:** Doaa A. Abdulwahab, Mohamed A. El-Missiry, Sameh Shabana, Azza I. Othman, Maggie E. Amer

**Affiliations:** Faculty of Science, Mansoura University, Mansoura, Egypt

**Keywords:** Melatonin, Heart, Pancreas, Oxidative stress, Inflammatory cytokines, Hyperglycemia

## Abstract

Cardiomyopathy and pancreatic injury are health issues associated with type 2 diabetes mellitus (T2DM) and are characterized by elevated oxidative stress, inflammation and apoptosis. Melatonin (MLT) is a hormone with multifunctional antioxidant activity. The protective effects of MLT on the heart and pancreas during the early development of diabetic cardiomyopathy and pancreatic injury were investigated in male Wistar rats with T2DM. MLT (10 mg/kg) was administered daily by gavage for 15 days after diabetic induction. Treatment of diabetic rats with MLT significantly normalized the levels of serum glucose, HbA1-c, and the lipid profile and improved the insulin levels and insulin resistance compared with diabetic rats, affirming its antidiabetic effect. MLT significantly prevented the development of oxidative stress and sustained the levels of glutathione and glutathione peroxidase activity in the heart and pancreas of diabetic animals, indicating its antioxidant capacity. Additionally, MLT prevented the increase in proinflammatory cytokines and expression of Bax, caspase-3 and P53. Furthermore, MLT enhanced the anti-inflammatory cytokine IL-10 and antiapoptotic protein Bcl-2. MLT controlled the levels of troponin T and creatine kinase-MB and lactate dehydrogenase activity, indicating its anti-inflammatory and antiapoptotic effects. Histological examinations confirmed the protective effects of MLT on T2DM-induced injury in the myocardium, pancreas and islets of Langerhans. In conclusion, the protective effects of melatonin on the heart and pancreas during the early development of T2DM are attributed to its antihyperglycemic, antilipidemic and antioxidant influences as well as its remarkable anti-inflammatory and antiapoptotic properties.

## 1 Introduction

Despite significant improvements in the past two decades, type 2 diabetes mellitus (T2DM)-induced cardiac complications present a major global challenge for human health at all ages. Cardiovascular diseases rank first among diabetic complications causing morbidity and mortality [1]. The Framingham study showed that diabetic men and women were, respectively, two and five times more likely to develop heart problems than nondiabetic subjects [2]. T2DM is a metabolic disorder characterized by increased resistance to insulin in glucose-sensitive tissues and dyslipidemia leading to hyperglycemia, prolonged inflammation, and oxidative stress [3]. At the advanced stage, diabetic cardiomyopathy (DCM) clinically presents as heart failure in T1DM and T2DM [4]; however, the early events during the development of T2DM-induced organ injury are not completely understood. Therefore, understanding the pathogenesis and its molecular mechanisms constitutes a cornerstone to overcome and ameliorate the complications.

Hyperglycemia initiates the overproduction of reactive oxygen species (ROS) through several pathways [5]. Overproduction of free radicals is a feature of diabetes linked with diabetic heart diseases. Oxidative stress is a main factor in hyperglycemia-induced tissue injury and is responsible for events related to the early development of T2DM-induced organ injury [6]. Besides oxidative stress, inflammation and apoptosis resulted from hyperglycemia, hyperlipidemia and insulin resistance, which produce reactive oxygen and nitrogen species (ROS & RNS), are key players involved in diabetic heart injury [7]. Mitochondria are the main sites of superoxide radical production resulting from the leakage of electron flux in the respiratory chain. Mitochondrial dysfunction and oxidative stress are the main triggers of apoptosis, inflammation and the damage of cardiomyocytes, pancreatic cells [8] and several body organs. Diabetes-induced impairment of the redox balance in heart and pancreas was reported to be a crucial in the stimulation of pathological inflammation and DM complications. The hallmark of DCM is interstitial fibrosis and myocardial hypertrophy [9, 10].

A study have highlighted the important role of proinflammatory and anti-inflammatory cytokines in DCM [11]. Because oxidative stress is an important contributor in the pathogenesis of DCM, it is anticipated that preventing oxidative stress and mitochondrial dysfunction by antioxidant intervention in diabetic patients can reduce inflammation and improve redox homeostasis in the heart and pancreas [12].

Melatonin (5-methoxy-N-acetyltryptamine; MLT) is a strong endogenous antioxidant formed and secrete mainly by the pineal gland and decreases with age [13]. MLT is involved in the regulation of several physiological activities associated with the light-dark daily cycle. This antioxidant hormone shows daily rhythm in the pineal gland, plasma and pancreas, and low MLT levels in diabetes responsible for disturbed daily rhythms and decreased antioxidative capacity of tissues [14]. The plasma concentrations of MLT were lower in diabetic than healthy rats and humans [15]. In addition to its high antioxidant capacity, MLT exerts anti-apoptotic and anti-inflammatory effects under several circumstances, including obesity [16, 17, 18].

Recently, it is reported that the biochemical markers of the heart function were deceased after treatment with MLT in rats with myocardial ischemic-reperfusion injury [19]. This was attributed to its antioxidant actions and enhanced autophagy in cardiomyocytes [20]. Furthermore, MLT protected against left ventricular dysfunction after myocardial infarction through its remarkable antioxidant properties [21].

Blood glucose level stimulates pancreatic B-cells to secret insulin. Up-regulation of oxidative stress is associated hyperglycemia and responsible for the development of pancreatic beta-cell dysfunction in diabetes [22]. This is attributed to low antioxidant capacity of pancreatic beta-cells of Langerhans which is more vulnerable to oxidative damage than other body cells [23]. MLT influences metabolic disorders including diabetes via the control of insulin release in vivo and in vitro [24]. MLT regulates the hormone secretion by pancreatic islets of specific, G-protein-coupled melatonin receptor types MT1 and MT2 on main pancreatic islet cells including Beta-cells [25]. Recently, it is reported that MLT increased proliferation of pancreatic cells through activation of MT2 in vitro [26].

The information on the protection against the pathophysiological process during the early development of T2DM-induced DCM is not fully clear and needs to be specified in the heart and pancreas. This study investigated the effect of MLT on hyperglycemia that influences the redox state, inflammation and apoptosis in the heart and pancreas of T2DM-induced rats at the early phase of the development of T2DM.

## 2 Materials and methods

### 2.1 Chemicals

Melatonin and streptozotocin were purchased from Sigma Chemical Company (St. Louis, MO, USA). Other laboratory chemicals were obtained from local sources.

### 2.2 Animals

Adult male Wister rats (8 weeks old) with body weight 170–200 g were purchased from the Egyptian Vaccine Company (VACERA, Cairo, Egypt). All rats were maintained under standard laboratory conditions at 25 °C with 12-h alternating light and dark cycles. The rats were allowed for commercial rodent standard diet and water ad libitum. The experimental protocol for the treatment of rats was performed according to guidelines and approved by the Institutional Animal Ethics Committee of Mansoura University (Sci-Z-Ph-2020-16).

#### 2.2.1 Induction of the experimental T2DM model

Overnight-fasted rats were injected with nicotinamide (100 mg/kg, ip) 20 min before streptozotocin (STZ) (55 mg/kg) to induce T2DM. Physiological saline was used for preparation of nicotinamide while STZ was prepared in fresh citrate buffer (0.1 M, pH: 4.5) [27]. After three days, the glucose levels in blood were assessed using glucometer (Elegance CT-X10, GmbH & Co. KG, Germany). The rats with fasting blood glucose levels above 250 mg/dl denote development of hyperglycemia and were selected for the experiment.

### 2.3 Experimental design and treatment

**Figure 1:**
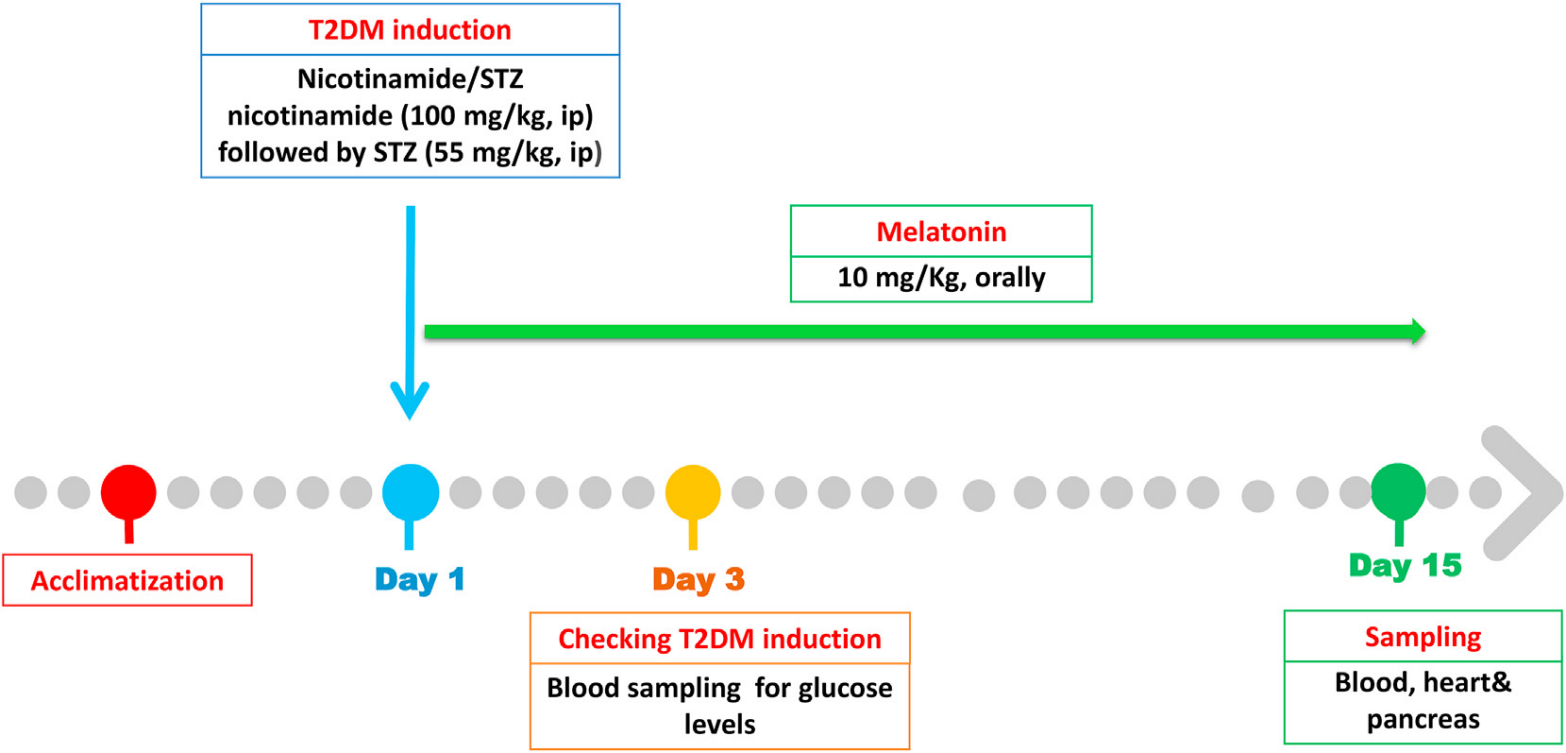
Diagram of the experimental design.

After the acclimatization period, the animals were divided into four groups, 5 animals in each group as follows (Figure 1): in the control group (Cont) the rats received standard diet and tap water only. In the melatonin treated group (MLT), rats were given MLT (10 mg/kg) by stomach tube daily for 15 days between 10:0 and 11:00 am [28]. The 3^rd^ group was diabetic group (DM) in which the rats were treated with single dose of nicotinamide following STZ [25] to develop diabetes. In the fourth group, rats were injected with MLT at 3^rd^ day after induction of diabetes for 15 days. The experimental period was 15 days post diabetic induction.

The blood glucose levels of all animals were monitored every five days during the experimental period by glucometer.

### 2.4 Sample collection

Blood samples from all rats were collected from hearts under ketamine/xylazine (0.1 ml/100 g, i.p.) anesthesia into nonheparinized tubes. Sera were separated after centrifugation at 3000 rpm for 15 min and stored at −20 °C for biochemical determination. The rats then were decapitated and the heart and pancreas were obtained then cleaned. For biochemical analysis, portions of the heart and pancreas were homogenized and centrifuged. The supernatant was obtained and stored at −20 °C for biochemical assay in tissue. For histological, histochemical and immunohistochemical studies, portions of both tissues were fixed in 10 % neutral formalin until processing.

### 2.5 Biochemical analysis

The levels of serum glucose and Hb1c in the blood were evaluated using ready-made kits provided by Spinreact (Girona, Spain) and BioSystems (Barcelona, Spain), respectively. The levels of serum insulin was assayed using the ELISA kit (RayBiotech, Georgia, USA; Catalog #: ELR-Insulin) with a detection range (5 uIU/ml - 300 uIU/mL). The homeostasis model of insulin resistance (HOMA-IR) was calculated using the following formula (1):

(1) $$\text{ HOMA}-\text{IR}=\frac{\left(\text{fasting insulin }(\text{μIU}/\text{mL})\times \text{ fasting glucose }(\text{mg}/\text{dL}\right)}{405}$$

Total cholesterol (TC), triglyceride (TG) and high-density lipoprotein cholesterol (HDL-C) were assayed according to the manufacturer's instructions (Spinreact, Girona, Spain). Low-density lipoprotein cholesterol (LDL-C) and very low-density lipoprotein cholesterol (VLDL-C) levels were determined using Friedewald's formula (2 &3) [29].

(2) LDL-C = TC − (HDL-C + VLDL-C)

(3) VLDL-C = TG/5

Interleukin 6 (IL-6; Catalog #: EK0412) with a detection range (62.5pg/ml-4000 pg/ml), interleukin 1 beta (IL-1b; Catalog #: EK0393) with a detection range (31.2pg/ml-2000 pg/ml), interleukin 10 (IL-10; Catalog #: EK0418) with a detection range (31.2pg/ml-2000 pg/ml), tumor necrosis factor alpha (TNF-α; Catalog #: RTFI01177) with a detection range (15.625–1000 pg/ml) and troponin T (Catalog #: KT-30669) with a detection range (0.078–5 ng/ml) in serum were assessed using kits provided by BosterBio (California, USA), ElisaGenie (London, UK) and Kamiya Biomedical (Seattle, USA), respectively. The activities of creatine kinase (CK-MB) and lactate dehydrogenase (LDH) in serum were determined using kits provided by BioSystems (Barcelona, Spain). Lipid peroxidation was determined by estimating the amount of 4-hydroxynonenal (4-HNE) according to the manufacturer's instructions (FineTest, Wuhan, China) with a detection range (31.25–2000 pg/ml) in both heart and pancreatic tissues. The glutathione (GSH) concentration and glutathione peroxidase (GPx) activities were estimated using kits supplied by Biodiagnostic (Dokki-Giza, Egypt) in both heart and pancreatic tissues. The activities of aspartate aminotransferase (AST), alanine aminotransferase (ALT), as well as the contents of albumin, total bilirubin and total protein in the serum, were estimated using kits provided by Biodiagnostic (Dokki-Giza, Egypt).

### 2.6 Detection of P53, Bax, caspases-3 and Bcl-2

Paraffin-embedded sections of the heart and pancreas were deparaffinized in xylene and then processed for immunohistochemical staining using the labeled streptavidin-biotin immunoperoxidase technique [30]. The mouse monoclonal anti-p53 antibody (DO-1: sc-126) was obtained from Santa Cruz, CA, USA; diluted 1:50). The rabbit polyclonal primary antibodies for caspase-3 (ab13847), Bax (ab53154) and Bcl-2 (ab59348) were obtained from Abcam, Cambridge, MA, USA. The dilution was 1:500 for caspase-3 and 1:50 for Bax and Bcl-2. The sections incubated with the primary antibodies overnight at 4 °C. The labeling index was assessed as described previously [31] using Image J software [32].

### 2.7 Histological investigation

Samples of the heart and pancreas tissues were subjected to standard procedures, in which the specimens were fixed, dehydrated, cleared and then embedded in paraffin wax. Next, 5-μm sections were prepared and stained with hematoxylin and eosin (H&E). The stained sections were observed using a light microscope and then were photographed using Olympus light microscope with camera (Amscope MU1000). Digital morphometric study for heart and pancreas was performed using computer assisted digital image analysis for quantification.

### 2.8 Detection of cardiac fibrosis

The sections of the heart were deparaffinized then were stained with Masson's trichrome. The stained sections were examined and photographed using an ordinary light microscope. The collagen fibers were stained blue relative to the extent of fibrosis. Quantification of fibrosis in heart tissue was performed using computer assisted digital image analysis.

### 2.9 Statistical analysis

All the data were statistically performed using GraphPad Prism 6.0 software after checking the normality distribution for each group. The results are expressed as the means ± standard error of the mean (SEM) (n = 5). Statistical comparisons were evaluated by one-way analysis of variance (ANOVA) followed by Duncan's multiple range tests. The percent of change in the treated groups was calculated compared with that in the control.

## 3 Results

### 3.1 MLT improves the glucose and HbA1c levels and insulin resistance

**Figure 2:**
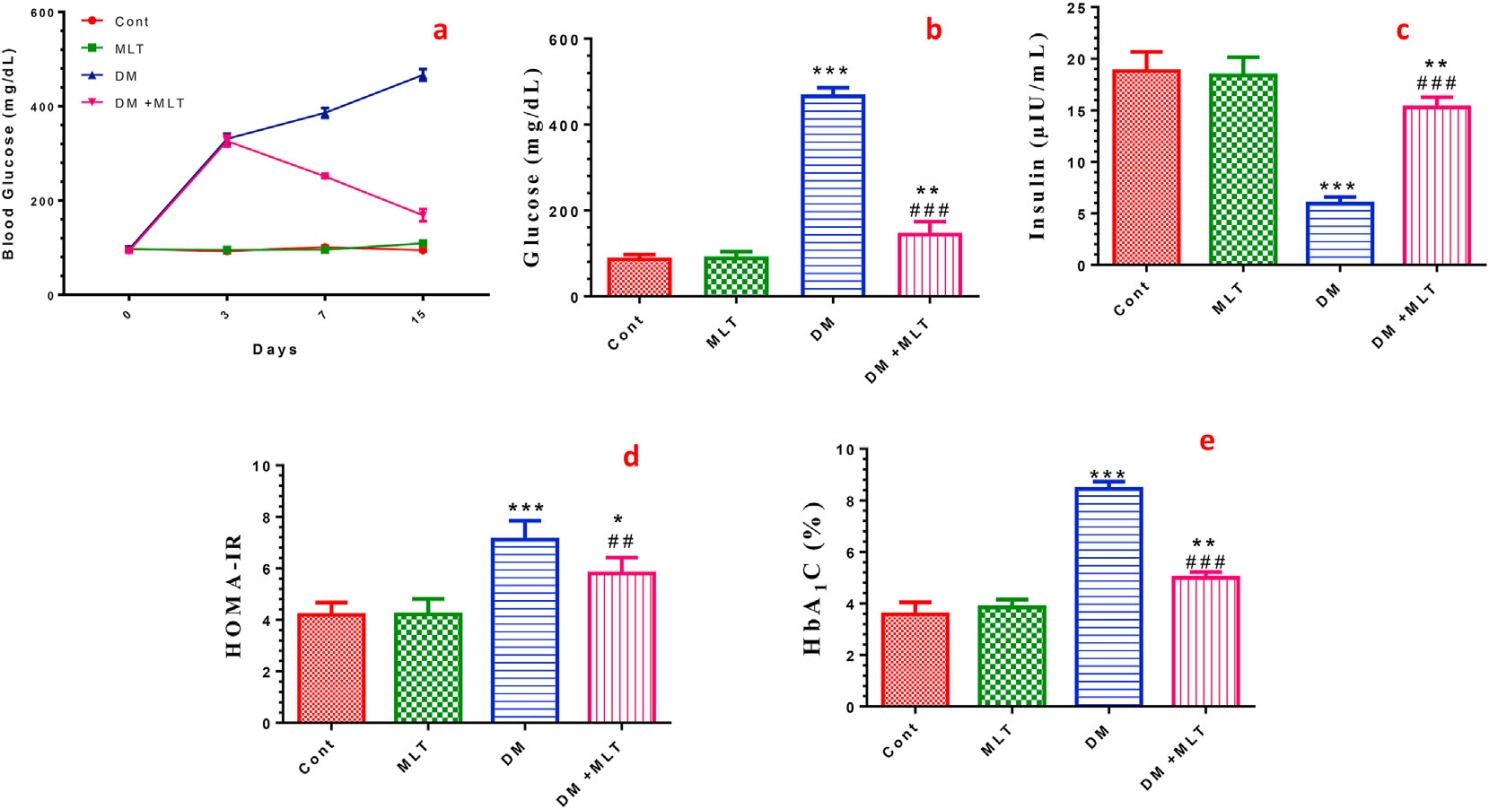
Effect of diabetes (DM) and melatonin (MLT) on the fasting blood glucose levels at the indicated time points (a), serum glucose (b), insulin (c), insulin resistance index (HOMA-IR) (d) and glycosylated hemoglobin (HbA1C) (e) levels of rats in the different groups. Values are expressed as means ± SEM; (n = 10) for fasting blood glucose and n = 5 for other parameters. (∗, ∗∗ and ∗∗∗ indicate statistical significance at P < 0.05, P < 0.01 and P < 0.001, respectively, compared to the control group. ## and ### indicate statistical significance at P < 0.01 and P < 0.001, respectively, compared to the diabetic group).

T2DM rats showed a significant increase in the glucose and HbA-1c levels 15 days after diabetic induction, indicating the development of hyperglycemia compared with the control rats. Diabetic rats showed a significant decrease in insulin levels and high HOMA-IR compared with the control animals, indicating the development of insulin resistance. Treatment of diabetic rats with MLT significantly prevented the increase in the blood levels of glucose, HbA-1c and insulin compared with untreated diabetic rats, but the levels were still higher than those of the control group. Consequently, HOMA-IR was significantly improved compared with that in the untreated diabetic rats and showed values near the control levels (Figure 2, S1). Treatment with MLT altered neither the glucose or HbA1c levels nor insulin resistance in T2DM rats compared with those in normal rats.

### 3.2 MLT improves the serum lipid profile in T2DM

**Figure 3:**
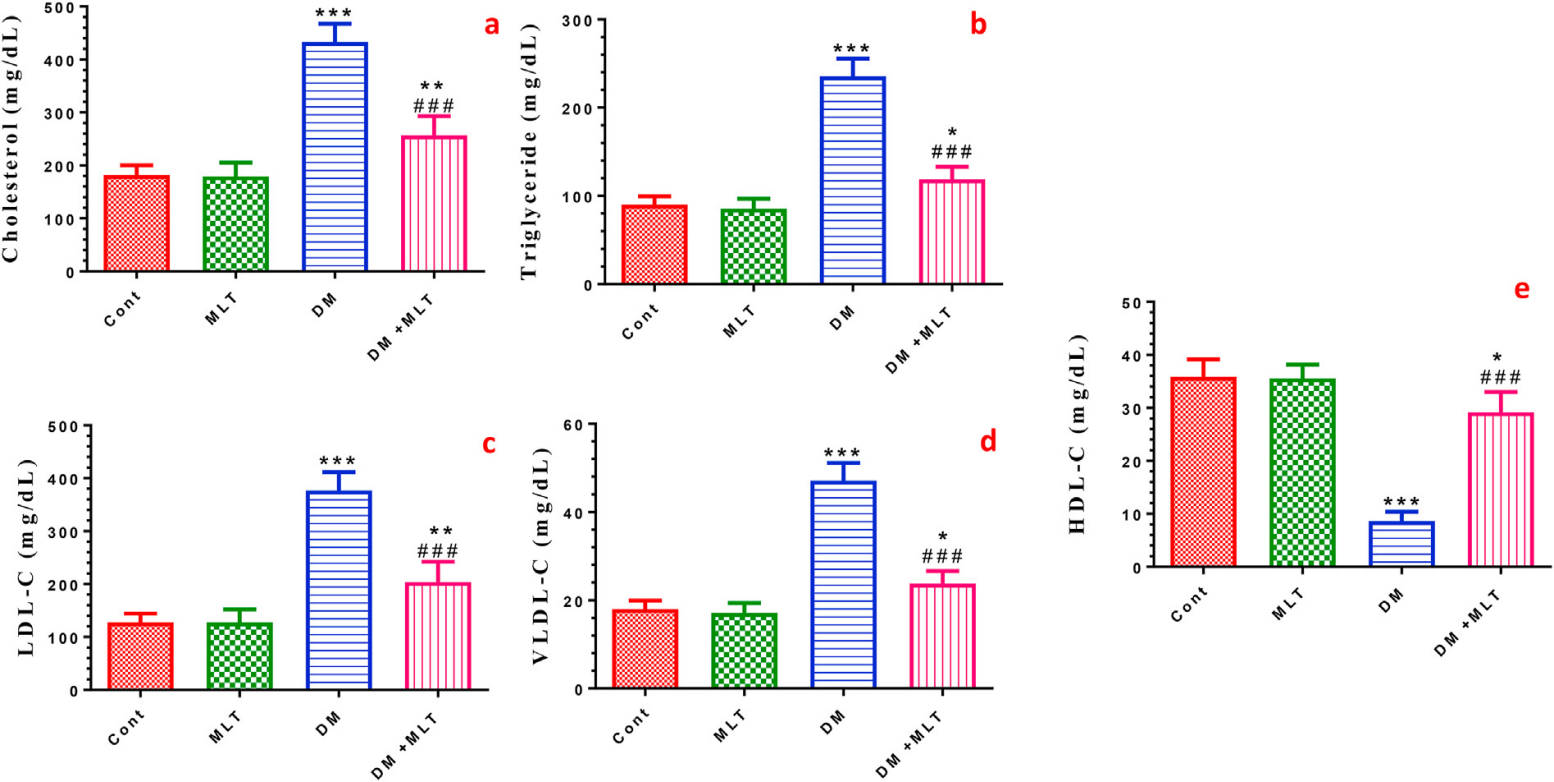
Effect of DM and MLT on the lipid profiles. Total cholesterol (a), triglyceride (b), low-density lipoprotein (LDL-C) (c), very low-density lipoprotein (VLDL-C) (d) and high-density lipoprotein (HDL-C) (e) serum levels of rats in the different groups. Values are expressed as means ± SEM; (n = 5). (∗, ∗∗ and ∗∗∗ indicate statistical significance at P < 0.05, P < 0.01 and P < 0.001, respectively, compared to the control group. ### indicate statistical significance at P < 0.001, respectively, compared to the diabetic group).

Diabetic rats showed a disrupted lipid profile presented by significant elevation of TC, TG, LDL-C and VLDL-C accompanied by a significant decrease in HDL-C levels in the serum of diabetic rats compared with those in control animals. Compared with untreated diabetic rats, MLT treatment ameliorated dyslipidemia in diabetic rats. The serum lipid profile was insignificantly changed in rats treated with MLT only (Figure 3, S2).

### 3.3 MLT ameliorates oxidative stress markers and improves the antioxidants levels in the heart and pancreas

**Figure 4:**
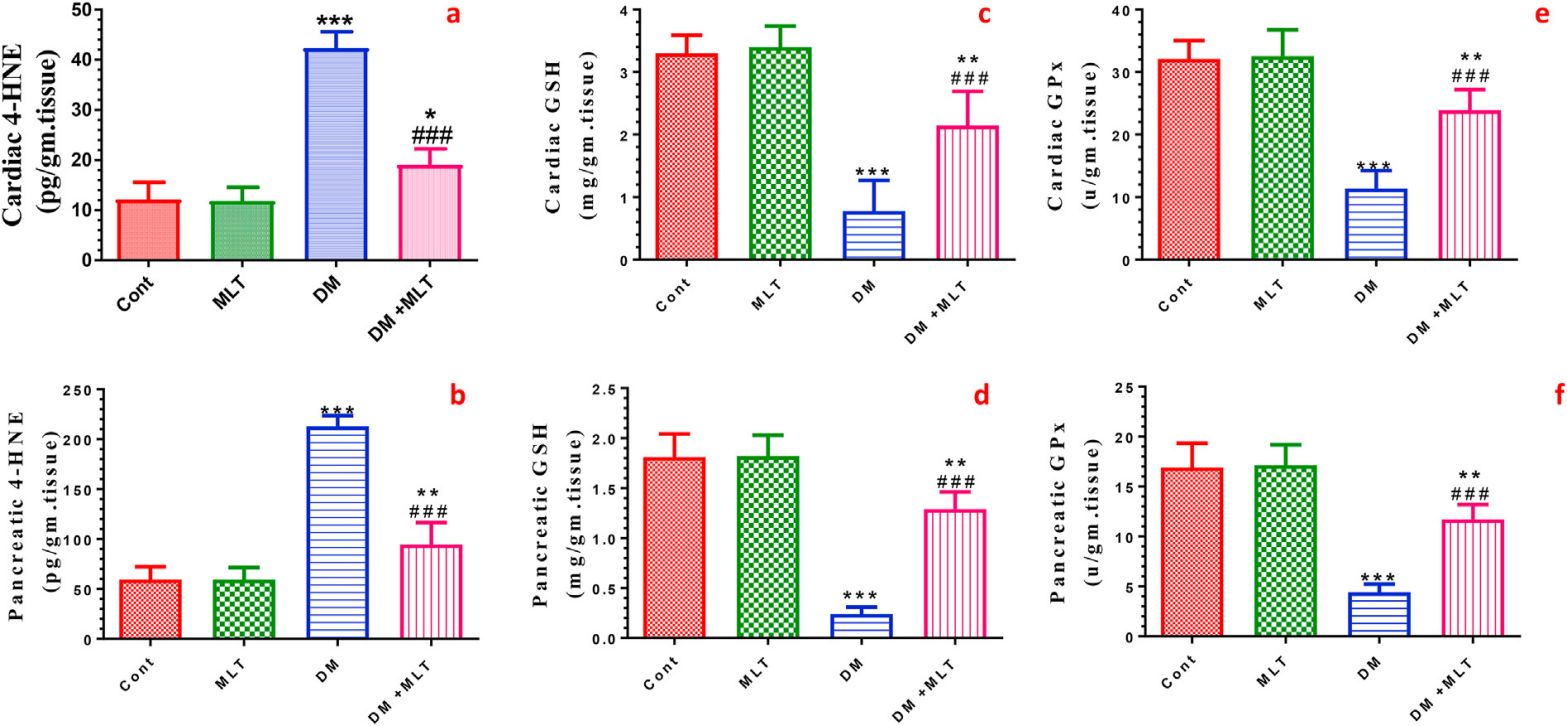
Effect of DM and MLT on the oxidative stress marker 4-hydroxynonenal (4-HNE) (a), antioxidant glutathione (GSH) content (c) and glutathione peroxidase (GPx) activity (e) in the heart tissues of rats in the different groups. Additionally, the effect of DM and MLT on the oxidative stress marker 4-hydroxynonenal (4-HNE) (b), antioxidant glutathione (GSH) content (d) and glutathione peroxidase (GPx) activity (f) in the pancreatic tissues of rats in the different groups. Values are expressed as means ± SEM; (n = 5). (∗, ∗∗ and ∗∗∗ indicate statistical significance at P < 0.05, P < 0.01 and P < 0.001, respectively, compared to the control group. ### indicate statistical significance at P < 0.001, respectively, compared to the diabetic group).

The effect of MLT treatment on the 4-hydroxynonenal (4-HNE) content was measured to evaluate lipid peroxidation in the heart and pancreas of all the studied groups. The heart and pancreas of diabetic rats showed a significant elevation in the 4-HNE level compared with those of the control animals. The increase in the 4-HNE level was significantly prevented by oral treatment with MLT compared with that in untreated diabetic rats. By contrast, a significant depletion in the GSH levels and GPx activity was demonstrated in the heart and pancreas of diabetic rats compared with that of control rats. Treatment of diabetic rats with MLT significantly prevented the decrease in the GSH and GPx levels in both tissues compared with untreated diabetic rats. Animals that received MLT alone showed insignificant changes in the 4-HNE and antioxidant levels compared with the normal control animals (Figure 4, S3).

### 3.4 MLT alleviates pro-inflammatory cytokines and improves anti-inflammatory cytokines

**Figure 5:**
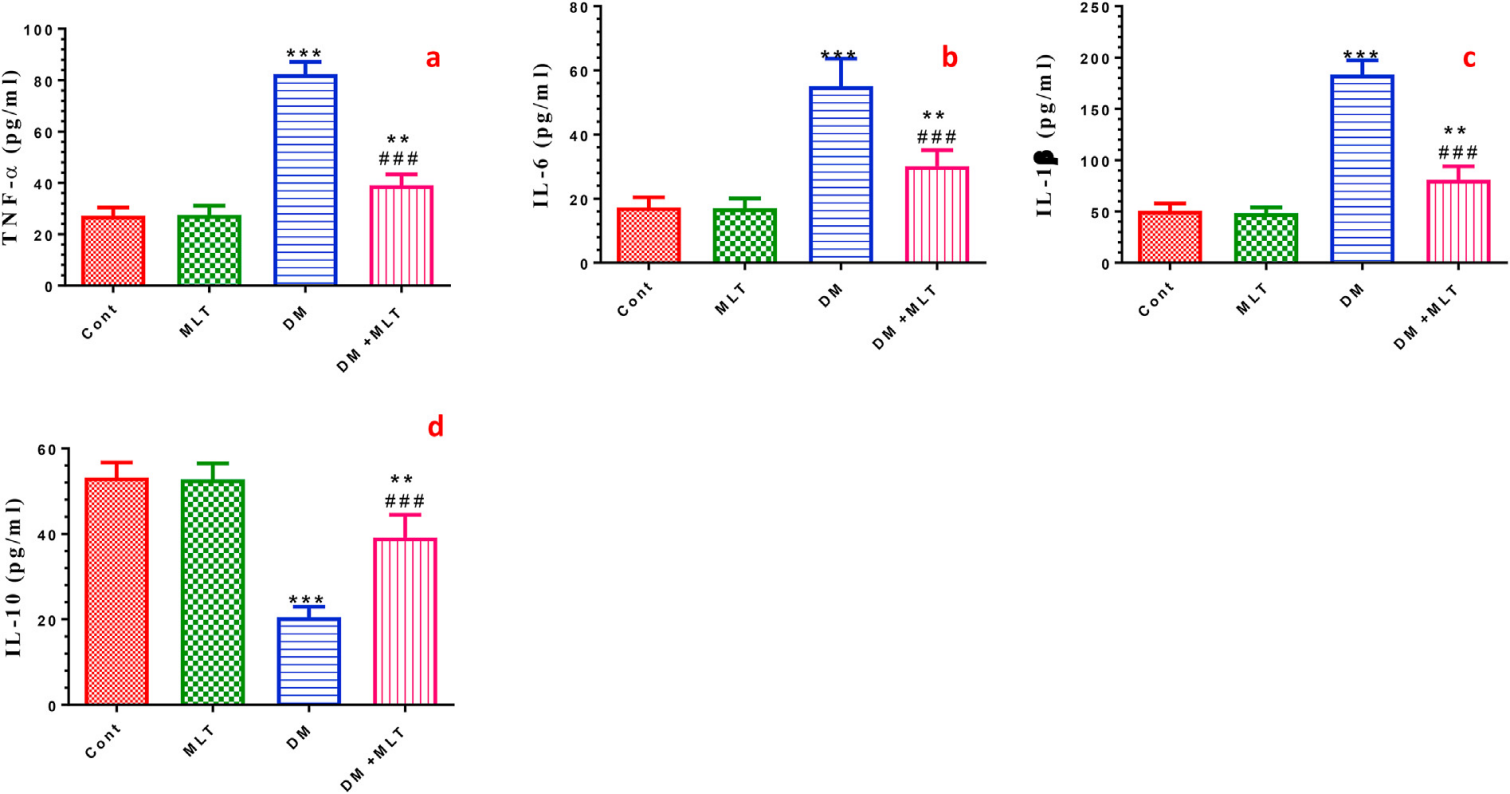
Effect of DM and MLT on the serum levels of pro-inflammatory mediators. Tumor necrosis factor alpha (TNF-α) (a), interleukin 6 (IL-6) (b), interleukin 1 beta (IL-1β) (c) and anti-inflammatory cytokine interleukin 10 (IL-10) (d) of rats in the different groups. Values are expressed as means ± SEM; (n = 5). (∗, ∗∗ and ∗∗∗ indicate statistical significance at P < 0.05, P < 0.01 and P < 0.001, respectively, compared to the control group. ### indicate statistical significance at P < 0.001, respectively, compared to the diabetic group).

Diabetic animals exhibited a significant surge of inflammatory cytokines, including TNF-α, IL-6 and IL-1β, with a significant decline in the anti-inflammatory cytokine IL-10 level in the serum of diabetic animals compared with that of the control group. Daily administration of MLT in diabetic rats for 15 days significantly decreased the concentrations of the inflammatory cytokines and elevated the levels of anti-inflammatory cytokine compared with diabetic rats. Treatment with MLT for 15 days showed insignificant changes in the serum levels of these cytokines in normal rats (Figure 5, S4).

### 3.5 MLT improves cardiac function biomarkers

**Figure 6:**
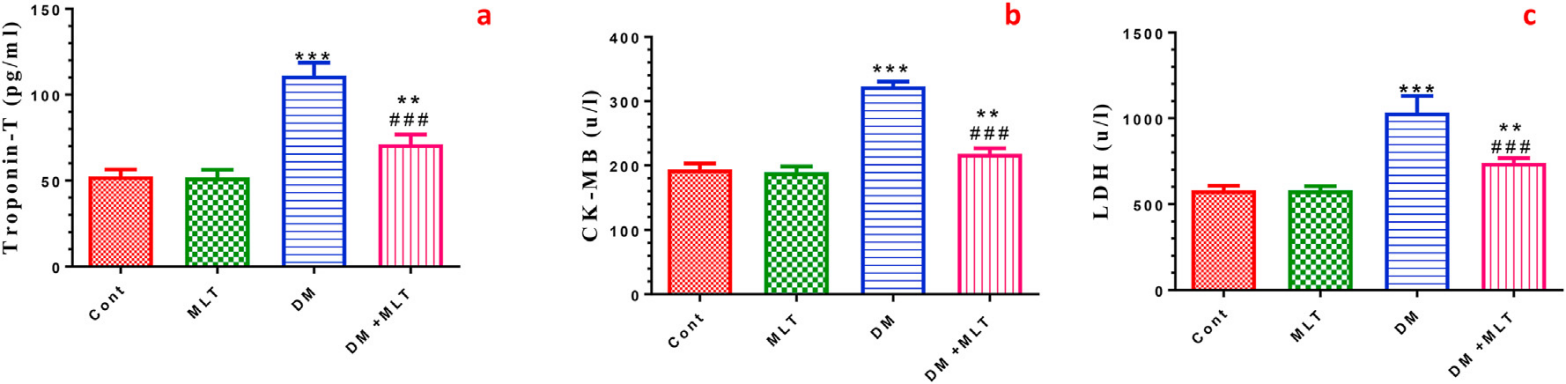
Effect of DM and MLT on the serum Troponin-T level (a) and cardiac biomarker enzymes creatine kinase myocardial band (CK-MB) (b) and lactate dehydrogenase (LDH) (c) of rats in different groups. Values are expressed as means ± SEM; (n = 5). (∗∗ and ∗∗∗ indicate statistical significance at P < 0.05, P < 0.01 and P < 0.001, respectively, compared to the control group. ### indicate statistical significance at P < 0.001, respectively, compared to the diabetic group).

To evaluate the heart function the levels of troponin-T and the activities of CK-MB and LDH were assessed in serum. These parameters were significantly increased in the serum of diabetic rats compared with that of the control group. The administration of MLT to diabetic rats significantly prevented the elevation of these biochemical biomarkers compared with untreated diabetic animals. Furthermore, treatment with MLT only showed an insignificant alteration in these biomarkers in normal rats (Figure 6, S5).

### 3.6 MLT ameliorates serum clinical biomarkers in the T2DM rat model

**Figure 7:**
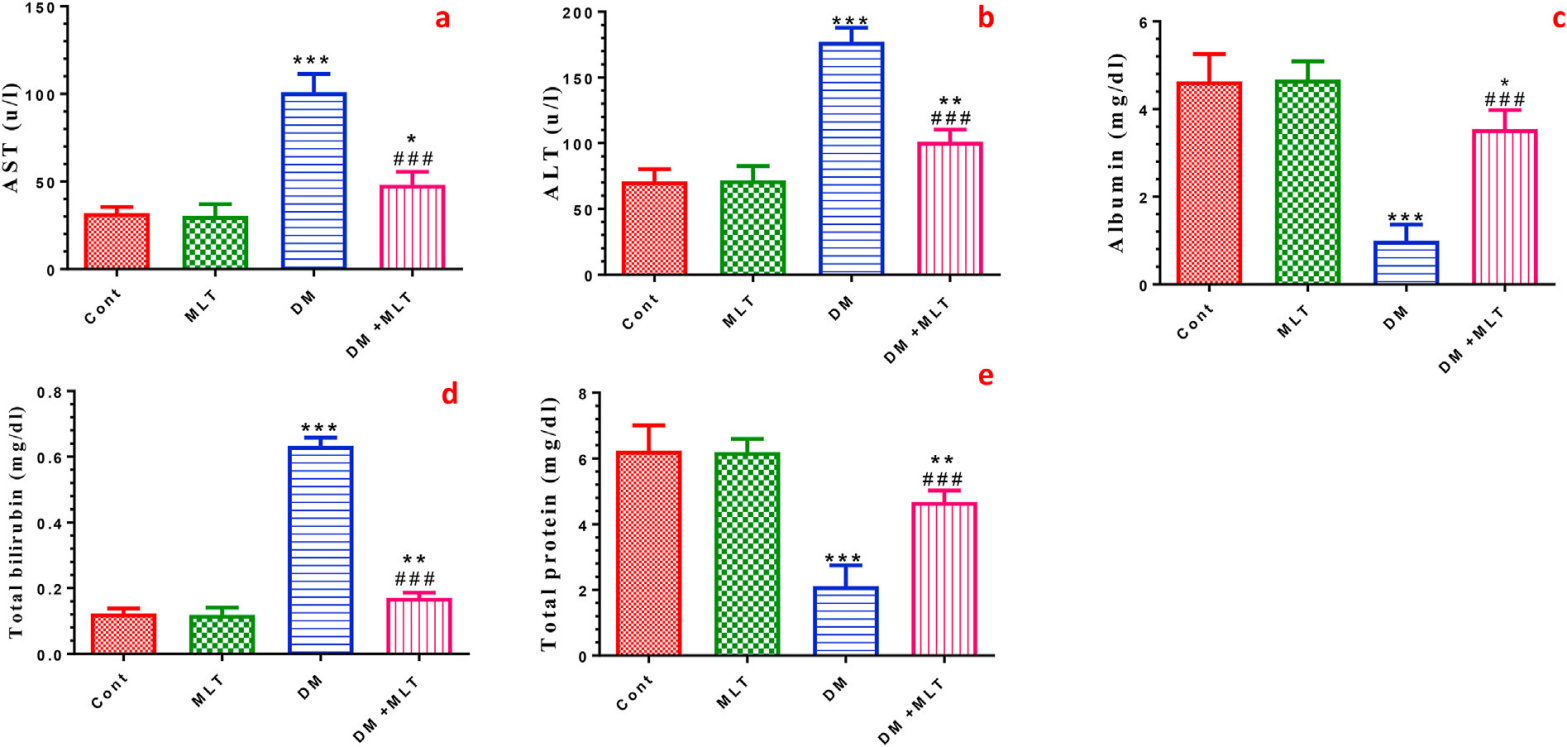
Effect of DM and MLT on the serum levels of liver function enzymes. Aspartate aminotransferase (AST) (a), alanine aminotransferase (ALT) (b), albumin c, total bilirubin (d) and total protein (e) of rats in the different groups. Values are expressed as means ± SEM; (n = 5). (∗, ∗∗ and ∗∗∗ indicate statistical significance at P < 0.05, P < 0.01 and P < 0.001, respectively, compared to the control group. ### indicate statistical significance at P < 0.001, respectively, compared to the diabetic group).

The induction of T2DM exerted a significant increase in the serum levels of liver function enzymes, including ALT and AST, as well as the total bilirubin content, with a significant decrease in the serum levels of albumin and total protein compared with those in the control group. Compared with untreated diabetic rats, administration of MLT into diabetic rats showed a significant improvement the in the levels of these biomarkers in serum. Treatment with MLT only showed an insignificant alteration in these biomarkers in normal rats (Figure 7, S6).

### 3.7 MLT normalizes apoptosis-regulating proteins

**Figure 8:**
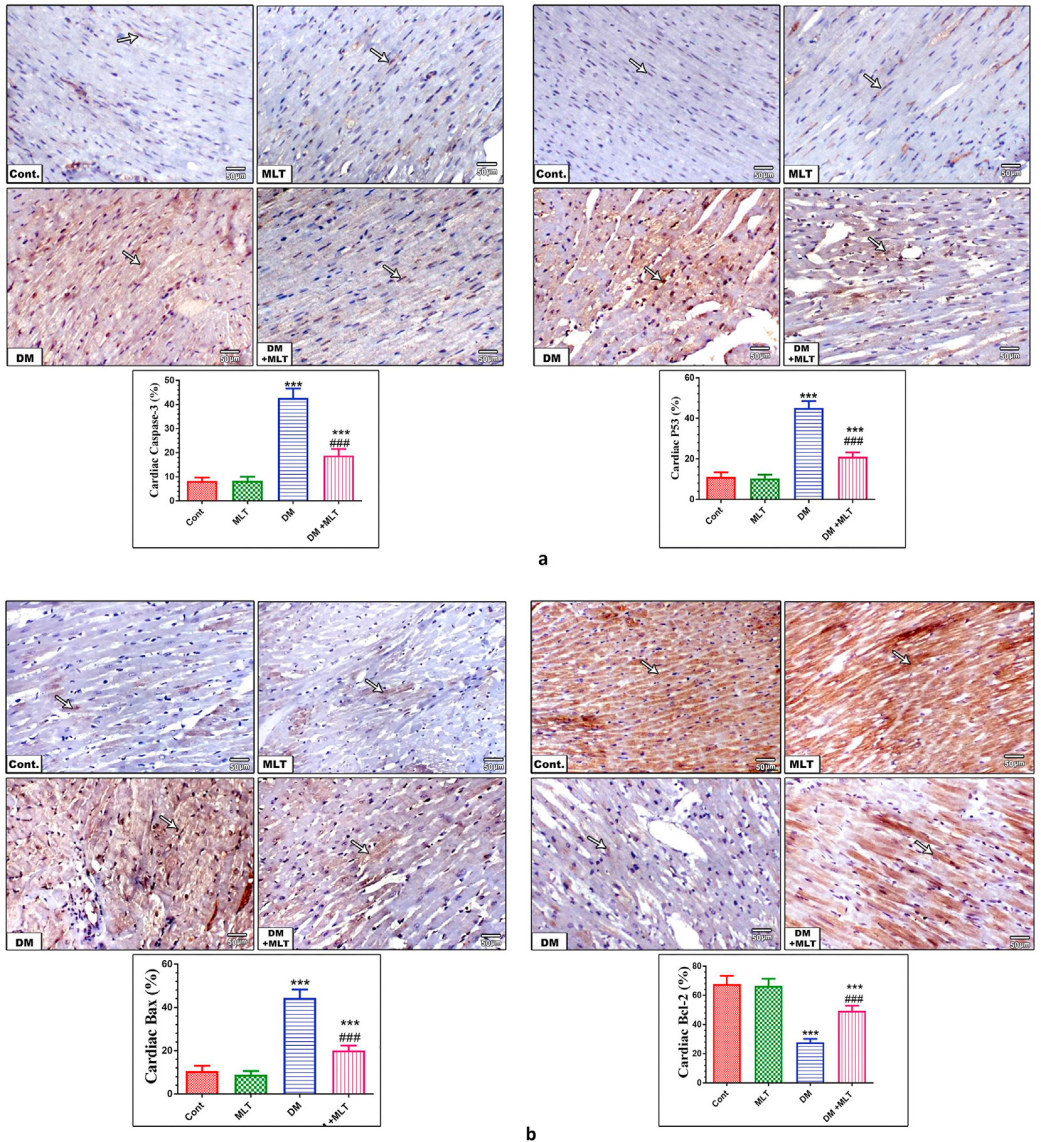
a. Expression levels of caspase-3 and p53 in the heart in different animal groups. Control (Cont) rats showing mild expression of caspase-3 and p53 (arrows) within myocardial cells. The MLT group showed weak caspase-3 and p53 immuno-expression (arrows) in myocardial cells. Diabetic (DM) animals show marked immuno-expression of both caspase-3 and p53 (arrows) in myocardial cells respectively. Diabetic animals treated with MLT (DM + MLT) displayed a marked reduction in caspase-3 and p53 expression to almost normal (arrows) within myocardial cells (IHC, ×200). The values are expressed as the means ± SEM of 5 microscopic fields/tissue samples of caspase-3 and p53 immuno-expression. (∗∗∗ and ### indicate statistical significance at P < 0.001, compared to the control group and diabetic group respectively). b. Expression levels of Bax and Bcl-2 in the heart in different animal groups. Control (Cont) rats showing mild expression of Bax and marked immuno-expression of Bcl-2 (arrows) within myocardial cells. The MLT group showed weak Bax and marked Bcl-2 immuno-expression (arrows) in myocardial cells. Diabetic (DM) animals show marked immuno-expression of Bax and mild expression of Bcl-2 (arrows) in myocardial cells respectively. Diabetic animals treated with MLT (DM + MLT) displayed a marked reduction in Bax and strong Bcl-2 expression to almost normal (arrows) within myocardial cells (IHC, ×200). The values are expressed as the means ± SEM of 5 microscopic fields/tissue samples of Bax and Bcl-2 immuno-expression. (∗∗∗ and ### indicate statistical significance at P < 0.001, compared to the control group and diabetic group respectively).

**Figure 9:**
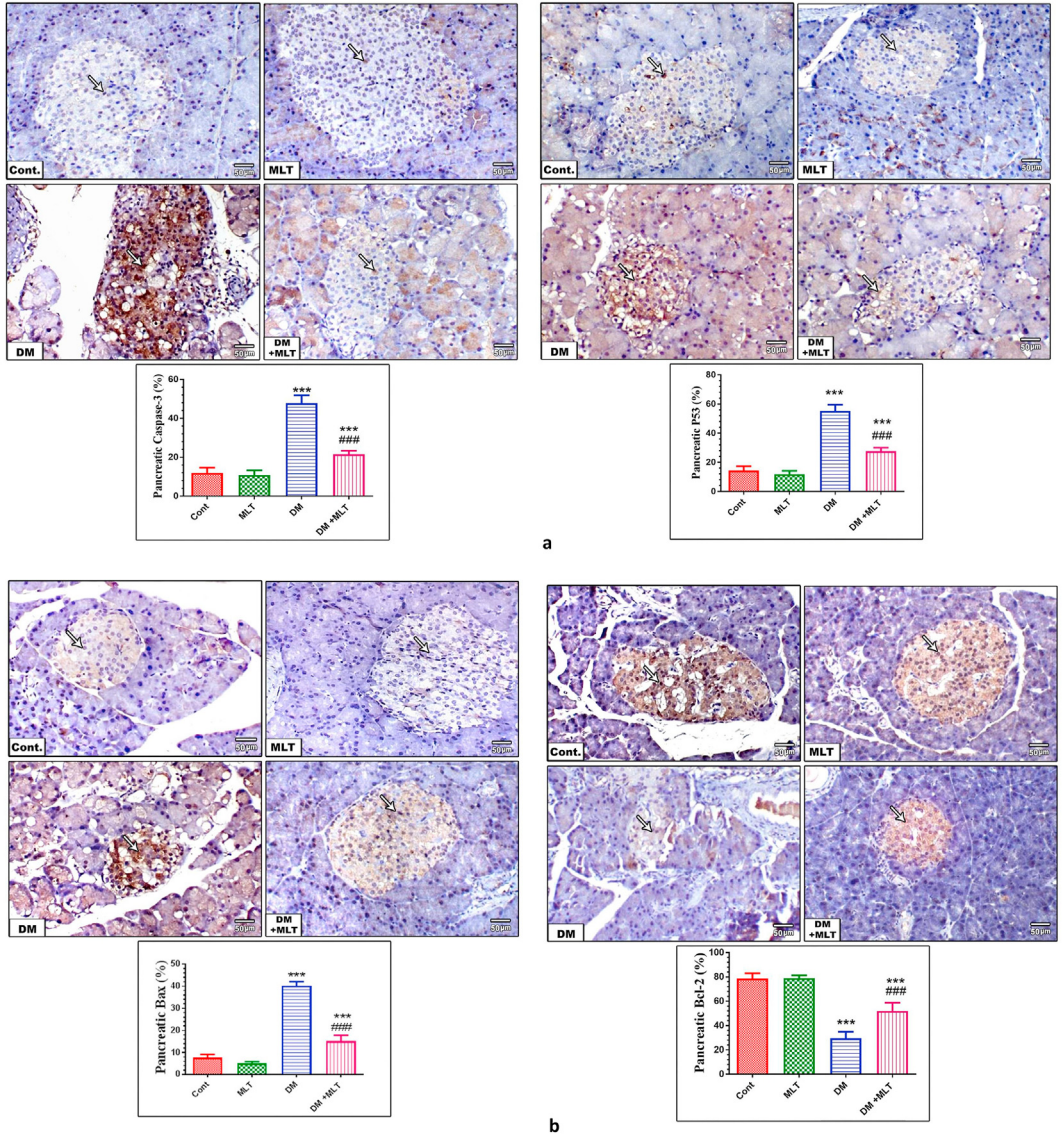
a. Expression levels of caspase-3 and p53 in the pancreas in different animal groups. Control rats displayed mild expression of caspase-3 and p53 (arrows) within pancreatic β-cells. The MLT group showed weak caspase-3 and p53 immuno-expression (arrows) in pancreatic β-cells. DM animals showed marked immuno-expression of both caspase-3 and p53 (arrows) in pancreatic β-cells. DM + MLT animals displayed a marked reduction of caspase-3 and p53 expression to almost normal (arrows) within pancreatic β-cells (IHC, ×200). The values are expressed as the means ± SEM of 5 microscopic fields/tissue samples of caspase-3 and p53 immuno-expression. (∗∗∗ and ### indicate statistical significance at P < 0.001, compared to the control group and diabetic group respectively). b. Expression levels of Bax and Bcl-2 in the pancreas in different animal groups. Control rats displayed mild expression of Bax and marked immuno-expression of Bcl-2 (arrows) within pancreatic β-cells. The MLT group showed weak Bax and strong Bcl-2 immuno-expression (arrows) in pancreatic β-cells. DM animals showed marked immuno-expression of Bax and mild expression of Bcl-2 (arrows) in pancreatic β-cells. DM + MLT animals displayed a marked reduction of Bax and marked Bcl-2 expression to almost normal (arrows) within pancreatic β-cells (IHC, ×200). The values are expressed as the means ± SEM of 5 microscopic fields/tissue samples of Bax and Bcl-2 immuno-expression. (∗∗∗ and ### indicate statistical significance at P < 0.001, compared to the control group and diabetic group respectively).

Immunohistochemical observation of the heart and islet of Langerhans tissues from control and MLT-only treated animals showed mild changes in caspase-3, p53, and Bax and marked expression of Bcl-2 within cardiomyocytes and the pancreas. By contrast, these tissues from diabetic animals showed marked expression of caspase-3, p53 and bax and a marked decrease in the expression of Bcl-2 within both tissues. The diabetic animals treated with MLT showed a marked improvement with a decrease in caspase-3, p53 and bax expression to almost normal levels in heart and islets of Langerhans tissues. This expression pattern was accompanied with a marked upregulation in the expression of Bcl-2 within both tissues. Treatment with MLT only for 15 days showed an insignificant alteration in the apoptotic biomarkers in normal rats (Figures 8 and 9, S7).

### 3.8 MLT protects against histopathological alterations in the heart and islets of Langerhans

**Figure 10:**
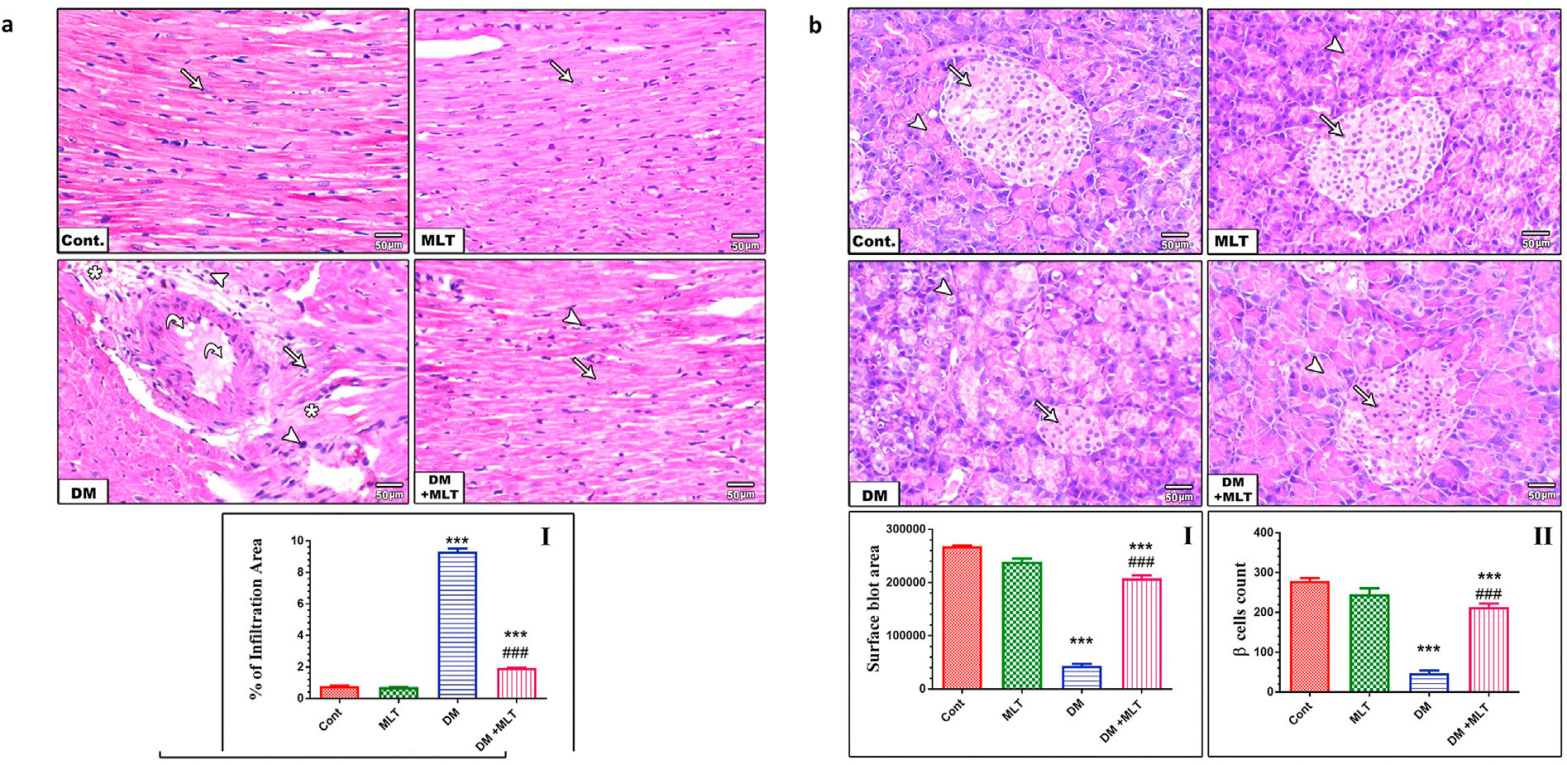
a. Hematoxylin- and eosin-stained cardiac sections of control and MLT-only-treated animals showing the regular arrangement of myocardial fibers with centrally located cigar-shaped nuclei (arrows). Diabetic rats (DM) showed disorganized arrangement of the myocardial fibers associated with perivascular myocardial degeneration (arrow), necrotic cardiac myocytes (asterisk) and mononuclear inflammatory cell aggregation (arrowhead) associated with vacuolation (curved arrow) and hyalinization in some of the cardiac myofibers. Diabetic rats treated with MLT (DM + MLT) showed improved arrangement of myocardial fibers associated with a mild degree of myocardial degeneration (arrow) and mild interstitial mononuclear inflammatory cell infiltration (arrow head), H&E, (× 200). The quantification of infiltration in heart sections of the different groups (I). Values are expressed as the means ± SEM. (∗∗∗ and ### indicate statistical significance at P < 0.001, compared to the control group and diabetic group respectively). b. Hematoxylin- and eosin-stained pancreatic sections of control and MLT-only-treated animals showing normal histological features of both exocrine and endocrine structures (arrow heads) indicates normal acinar structures and (arrows) indicates β-islets of Langerhans. Diabetic rats (DM) showed apoptosis of exocrine glands (arrowhead) and a marked shrunken in the size of the endocrine islets of Langerhans with a drastic decrease in the number of their cells (arrow). Diabetic rats treated with MLT (DM + MLT) showed mild degenerative changes within exocrine glands (arrowhead) and apparent increase in the islets of Langerhans size with normal histoarchitecture (arrow), H&E, (× 200). The quantification of islets of Langerhans blot area (I) and β cells count (II) within the blot area in sections of the pancreas of the different groups. Values are expressed as the means ± SEM. (∗∗∗ and ### indicate statistical significance at P < 0.001, compared to the control group and diabetic group respectively).

Histological observation of heart tissues from control and MLT only treated rats revealed a normal size and regular arrangement of the myocardial fibers, with centrally located cigar-shaped nuclei. By contrast, the heart sections of diabetic animals showed a distorted and an irregular wavy appearance of myocardial fibers associated with deeply stained pyknotic nuclei, cardiomyocyte degeneration and myofibrillar discontinuation, as well as interstitial mononuclear inflammatory cell infiltration. Additionally, the heart sections of diabetic animals treated with MLT reduced these changes and revealed a markedly mild degree of myocardial degeneration associated with mild interstitial mononuclear inflammatory cell infiltration (Figure 10a, Figure S8).

The pancreatic tissues of control and MLT-only treated rats exhibited a normal histological architecture of both exocrine and endocrine structures, represented by a normal appearance of β-islets of Langerhans areas surrounded by exocrine acinar structures. In the pancreas tissues of diabetic rats, degenerative and necrotic changes were consistently found within exocrine glands and a marked decrease in the size of the endocrine β-islets of Langerhans. However, pancreatic tissues of diabetic animals treated with MLT showed remarkable amelioration of histopathological alterations in the most of the islets of Langerhans cells with mild degenerative changes within exocrine glands (Figure 10b, Figure S9).

### 3.9 MLT mitigates cardiac fibrosis

**Figure 11:**
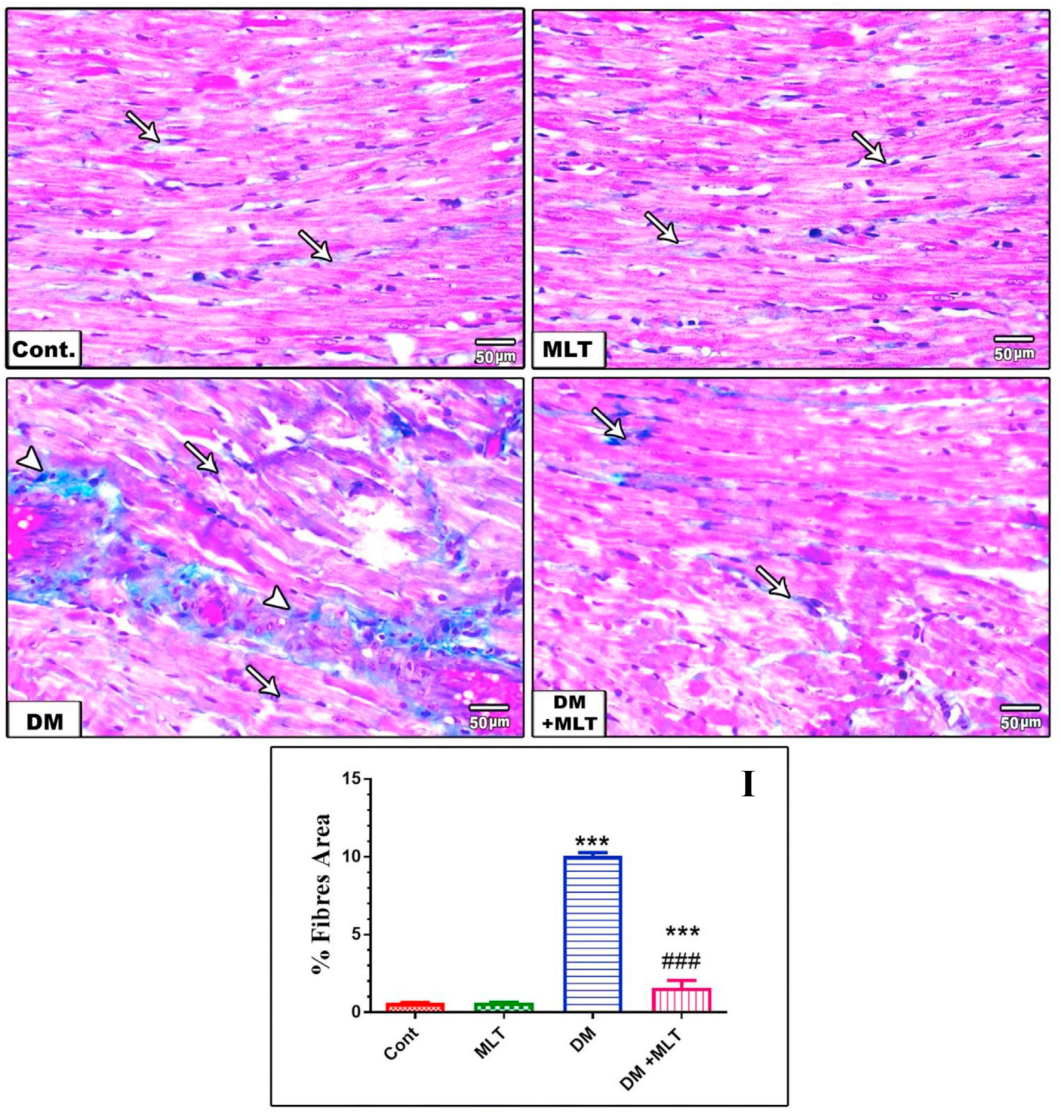
Masson trichrome-stained cardiac sections of control and MLT-only-treated animals showed normal thin interstitial collagen fibers in between myocardial fibers (arrow). (DM) rats showed marked perivascular (arrowhead) and interstitial (arrow) fibrosis. The (DM + MLT) group showed a marked decrease in interstitial fibrous connective tissue (arrows) (×200). Percent of fibrosis area in Masson trichrome stained heart sections in the different groups (I). Values are expressed as the means ± SEM. (∗∗∗ and ### indicate statistical significance at P < 0.001, compared to the control group and diabetic group respectively).

The control and MLT-only treated rats displayed scarce and ordinary spreading of collagen fibers, represented by thin interstitial fibrous connective tissue in the heart sections. T2DM-induced rats displayed a remarkable increase in collagen fiber deposition in the interstitial area of the heart. Treatment with MLT remarkably decreased the fibrosis in the heart of diabetic rats (Figure 11, Figure S10).

## 4 Discussion

Diabetes mellitus (DM) is a worldwide health issue, and patients with T2DM often develop complications, including cardiomyopathy and pancreatic cell injury. The multifunction of MLT has attracted pronounced attention as a potential agent for the prevention and treatment of various diseases [33]. The present study revealed that MLT improved hyperglycemia and protected the heart and pancreas against diabetic-induced injury, an effect attributed to antioxidant, anti-inflammatory and anti-apoptotic properties of melatonin.

Diabetic rats exhibited remarkable elevated concentrations of fasting blood glucose, HbA1c with increased insulin resistance, as indicated by the high HOMA-IR levels and decrease in the insulin level due to dysfunction in β-cells. Treatment with MLT significantly ameliorated hyperglycemia and the reduction of insulin levels, blocked the glycosylation of hemoglobin and improved insulin resistance in T2DM rats. These results corroborate the improvement of hyperglycemia by MLT treatment in several experimental models [3, 34, 35]. These findings agree with the ability of MLT to reverse the reduction of GLUT4 gene expression, glucose intolerance and insulin resistance, and augmented gluconeogenesis in MLT-deficient pinealectomized rats [36, 37]. The possible mechanism by which MLT exerts its effects could be mainly attributed to insulinogenic activity that stimulates insulin secretion, regenerate β-cells or even protect the remaining β-cells through its strong antioxidant properties [38]. The current data agree with previous study that MLT exhibited an anti-diabetic effect in STZ-induced T1DM in animals [28, 39]. Therefore, the present findings indicate that MLT can exert anti-hyperglycemic and insulin-sensitizing effects.

Furthermore, it was demonstrated that MLT exhibits an anti-dyslipidemic effect in rats with T2DM. A significant elevation in the serum triglyceride, total cholesterol, LDL-C and VLDL-C levels accompanied by a decrease in the serum HDL-C level were demonstrated in T2DM rats compared with the control group. Epidemiological and clinical studies have consistently demonstrated a positive correlation between hyperlipidemia as a result of diabetes and the incidence of cardiovascular diseases [40, 41]. Minimizing hyperlipidemia is a good approach to prevent and ameliorate cardiac diseases related to diabetes [42]. In the current study, the administration of MLT to diabetic rats caused a significant improvement in the lipid profile compared with the STZ-induced diabetic rats. These findings agree with those in which MLT improved the serum lipid fractions in serum of diabetic rats [43, 44, 45]. The mechanism underlying the lowering effect of MLT on cholesterol might be through decreasing the absorption of cholesterol from the intestine or increasing endogenous cholesterol clearance [46]. MLT efficiently prevented hyperlipidemia and enhanced HDL-C, which could be related to increased insulin secretion, which enhances lipid storage in adipocytes. Thus, the present study supports the anti-dyslipidemic effect of MLT that helps to protect against lipid-induced risk factors of cardiac diseases.

The effect of MLT on the oxidation of LDL and prevention of lipid peroxidation was reported in previous studies [47, 48]. It was suggested that hyperglycemia, increased ROS and the induction of oxidative stress are the main pathophysiological links between T2DM and the early progression of heart injury [49]. In the current study, the 4-HNE level, a lipid peroxidation product, was remarkably increased in the heart and pancreas and was significantly higher in T2DM rats than in the normal control rats, indicating an excessive oxidative stress state. The increased oxidative stress in diabetic rats may result from the continuous generation of ROS produced by hyperglycemia and glucose autoxidation [50, 51]. This expression is accompanied by a significant decrease in the GSH content and GPx activity in cardiac and pancreatic tissues. The disrupted redox homeostasis was improved by MLT treatment, as indicated by the lower 4HNE level and higher antioxidant levels in the heart and pancreas than in the untreated diabetic rats. This finding indicates the potent antioxidant ability of MLT by enhancing the GSH/GPx system. MLT scavenges ROS and RNS and increases antioxidant defenses, actions that prevent tissue damage, block transcriptional factors of pro-inflammatory cytokines and reduce free radical damage to biomolecules. Consequently, the restoration of redox balance can potentially contribute to the protection against T2DM-induced oxidative damage in the heart and pancreas [52, 53]. Myocardial inflammation has also been implicated in the pathophysiology of diabetic cardiomyopathy [54]. Inflammation is the main pathogenic feature and is associated with hyperlipidemia and hyperglycemia [55]. In cardiomyocytes, inflammatory signaling usually occurs as an early response to myocardial injury, which involves the overproduction of ROS from mitochondria [56]. Because nuclear factor-κB (NF-κB) is a key regulator of inflammatory responses, it up-regulates the expression of pro-inflammatory cytokines in the heart [55]. The latter authors claimed that it is unclear whether myocardial inflammation is involved in cardiac dysfunction in the experimental models of T2DM. In the current study, the serum concentration of TNF-α, IL-6 and IL-1β were significantly increased while IL-10 was significantly decreased in diabetic group, indicating an increased inflammatory reaction and a disrupted immune system. These results agree with the earlier reports that pro-inflammatory cytokines are directly responsible for the induction of diabetic complications and heart diseases [57]. The current data showed that treatment of diabetic rats with MLT resulted in a significant reduction in the levels of TNF-a, IL-1b and IL-6 and, importantly, the upregulation of the anti-inflammatory cytokine, IL-10. These results support the anti-inflammatory activity of melatonin against diabetes-induced heart and pancreas injury.

The histopathological observations of the heart and pancreas confirmed the aforementioned findings. The sections of the heart from diabetic rats showed remarkable damage in the form of necrosis of cardiac myocytes, vacuolation, hyalinization and interstitial mononuclear inflammatory cell infiltration in the heart tissue. Additionally, the present results demonstrated interstitial fibrosis in the heart tissue of diabetic rats that can result in the impairment of both diastolic and systolic functions [55]. These pathological changes agree with previous study showing the harmful impact of oxidative stress on the hearts of diabetic rats [27, 58]. These pathological alterations might be amenable to MLT treatment to reduce the risk of heart injury in the context of T2DM. Increased oxidative stress and elevated inflammatory cytokines contribute considerably to the histopathology and pathophysiology of diabetic heart injury, and the association of redox imbalance and diabetic cardiomyopathy provides a rational for antioxidant treatment for managing heart injury. Treatment with MLT, characterized by its potent antioxidant and anti-inflammatory properties, exerted remarkable protection of the heart histology and blocked fibrosis in the heart compared with the control, indicating an anti-fibrotic effect of MLT.

To further support the protective effect of MLT on the heart, we assessed some heart function parameters. A significant elevation in the activities of CK-MB and LDH and troponin-T levels was observed in the diabetic rats compared with those in control animals, indicating damage to the cardiomyocyte membranes that contributes to myocardial injury. These findings agree with that reported in previous studies [[59], [60]]. Treatment of diabetic rats with MLT prevented the elevation of cardiac function markers, indicating that MLT can sustain the integrity of cardiomyocytes and ameliorate myocardial injury. This might be attributed to its membrane-stabilizing property in T1DM [28]. Recent studies have shown that MLT sustains cardiomyocyte integrity and prevents membrane damage. Recently, it is reported that MLT sustains cardiomyocyte integrity and prevents the leakage of cardiac biomarkers in infarcted myocardium [61]. Furthermore, treatment with MLT significantly ameliorated the elevation of AST and ALT activities and total bilirubin concentrations and the decline in albumin and total protein contents in serum of diabetic rats. These findings are consistent with results reported by [[62], [63]], reflecting the efficacy and safety application of melatonin. Notably, these biochemical parameters were not altered in MLT only-treated rats, indicating that melatonin has a high safety profile [64].

The pancreatic tissue of diabetic rats exhibited histopathological alteration in which islets appeared shrunken in size and hypocellular with an altered histoarchitecture. These findings were consistent with those reported in previous studies [65, 66] and might be attributed to dysregulation of redox status, activation of inflammatory processes and apoptosis [67]. The current results showed that treatment of diabetic rats with MLT improved histopathological changes in pancreatic tissues compared with the untreated diabetic rats, indicating the potential protective effect of MLT and improvement of the pancreatic structure, including islets of Langerhans. The present results agree with other studies that the administration of MLT protected pancreas against oxidative damage and ameliorated histological damage, as well as the hyperamylasemia and hyperlipidemia via increasing antioxidant power and reduction of pro-inflammatory cytokines in cerulean-induced pancreatitis [68, 69]. These effects are supported by the current biochemical findings, including increased insulin secretion and amelioration of insulin resistance. The mechanism underlying the ameliorative effects of MLT was suggested to be through the antioxidant and hypoglycemic effects of MLT that protects pancreatic tissue [70].

To further reveal the mechanism underlying the improved heart and pancreas structures and function following treatment with melatonin, apoptosis-regulating proteins were investigated. A significant increase was found in the levels of pro-apoptotic proteins Bax, caspase-3 and p53 in both tissues of T2DM rats with decrease of the anti-apoptotic protein Bcl-2, indicating mitochondrial pathology. By contrast, treatment of diabetic rats with MLT restored the balance between regulating proteins of apoptosis. These findings indicate that MLT can protect the heart and pancreas by sustaining mitochondrial function and modulating the apoptosis process during the early onset of T2DM through its antioxidant and anti-apoptotic properties [15].

In the present study, MLT was used to ameliorate heart and pancreas injury induced by DM and to maintain their functions. Although there are several studies concerning glucose homeostasis, the effect of MLT on glucose homeostasis has not been clearly illustrated. This is due to incomplete information on how and where MLT exerts its action on glucose homeostasis. It is believed that the decreased glucose level in diabetic rat models is attributed to the restoration of pancreatic islet cells by ML [71]. Melatonin receptors are found in many cells, including pancreatic β-cells and cardiomyocytes, indicating the prevalent effects of MLT on several physiological functions [25]. It was reported that MLT protects pancreatic β-cells from STZ-induced cellular oxidative injury and the development of T2DM [72, 73].

Because mitochondrial dysfunction and diabetes are linked [74], mitochondrial pathology may play a key role in cardiac and pancreatic cell injury. Thus, targeting the mitochondrial integrity by MLT against oxidative damage is crucial for glucose homeostasis. Mitochondria are essential organelles for cellular function and contribute to the regulation of redox homeostasis, oxidation of free fatty acid and apoptosis [75]. Cardiomyocytes and pancreatic cells have a remarkable amount of mitochondria because of the high energy demand of these organs. Both hyperglycemia and hyperlipidemia can hyperpolarize mitochondria, resulting in enhanced ROS production [76, 77], explaining the mitochondrial pathology commonly observed in the critical organs of both types of diabetes.

The present results suggest that MLT protected the mitochondria function, as evidenced by the amelioration of the histological structure of both organs, restoration of redox balance and controlled release of the apoptotic regulating proteins in diabetic rats. As a cytoprotective agent, MLT has been reported as a mitochondrial protector due to its antioxidant and anti-inflammatory properties [78]. Experimental evidence reported that MLT can stabilize the mitochondrial functions, which could be participate in the prevention of progression of several diseases including pancreatitis [79]. Therefore, we suggest that MLT protects and sustains the mitochondrial function and integrity necessary for glucose homeostasis in T2DM.

In conclusion, treatment of T2DM with a pharmacological dose of MLT is a central approach to fortify the body's antioxidant defense system and consequently block hyperglycemia, oxidative stress, and inflammatory and apoptosis mechanisms, preventing and protecting against T2DM-induced complications at the early stage of organ injury.

## Declarations

### Author contribution statement

Doaa A Abdulwahab, Mohamed A El-Missiry, Sameh Shabana, Azza I Othman, Maggie E Amer: Conceived and designed the experiments; Performed the experiments; Analyzed and interpreted the data; Contributed reagents, materials, analysis tools or data; Wrote the paper.

### Funding statement

This research did not receive any specific grant from funding agencies in the public, commercial, or not-for-profit sectors.

### Data availability statement

Data included in article/supplementary material/referenced in article.

### Declaration of interests statement

The authors declare no conflict of interest.

### Additional information

No additional information is available for this paper.

## Appendix A Supplementary data

Supplementary legends Supplementary legends

S1 S1

S2 S2

S3 S3

S4 S4

S5 S5

S6 S6

S7 S7
