# Supplementary material for: Melatonin protects the heart and pancreas by improving glucose homeostasis, oxidative stress, inflammation and apoptosis in T2DM-induced rats
Source: Heliyon. 2021 Mar 12;7(3):e06474. doi: 10.1016/j.heliyon.2021.e06474 (PMC7970364; doi:10.1016/j.heliyon.2021.e06474)
Supplement: Supplementary legends [file mmc1.docx]

**S1** Share the Effect of diabetes (DM) and melatonin (MLT) on the fasting blood glucose levels at the indicated time points (A), serum glucose (B), insulin (C), insulin resistance index (HOMA-IR) (D) and glycosylated hemoglobin (HbA1C) (E) levels of rats in the different groups.

**S2** Share the Effect of DM and MLT on the lipid profiles. Total cholesterol (A), triglyceride (B), low-density lipoprotein (LDL-C) (C), very low-density lipoprotein (VLDL-C) (D) and high-density lipoprotein (HDL-C) (E) serum levels of rats in the different groups.

**S3** Share the Effect of DM and MLT on the oxidative stress marker 4-hydroxynonenal (4-HNE) (A), antioxidant glutathione (GSH) content (B), and glutathione peroxidase (GPx) activity (C) in the heart tissues of rats in the different groups. Additionally, the effect of DM and MLT on the oxidative stress marker 4-hydroxynonenal (4-HNE) (D), antioxidant glutathione (GSH) content (E), and glutathione peroxidase (GPx) activity (F) in the pancreatic tissues of rats in the different groups.

**S4** Share the Effect of DM and MLT on the serum levels of pro-inflammatory mediators. Tumor necrosis factor alpha (TNF-α) (A), interleukin 6 (IL-6) (B), interleukin 1 beta (IL-1β) (C) and anti-inflammatory cytokine interleukin 10 (IL-10) (D) of rats in the different groups.

**S5** Share the Effect of DM and MLT on the serum Troponin-T level (A) and cardiac biomarker enzymes creatine kinase myocardial band (CK-MB) (B) and lactate dehydrogenase (LDH) (C) of rats in different groups.

**S6** Share the Effect of DM and MLT on the serum levels of liver function enzymes. Aspartate aminotransferase (AST) (A), alanine aminotransferase (ALT) (B), albumin (C), total bilirubin (D) and total protein (E) of rats in the different groups.

**S7** Share the Effect of DM and MLT on the immune-expression levels of caspase-3 (A), p53 (B), Bax (C) and Bcl-2 (D) in the heart tissues of rats in the different groups. Additionally, the effect of DM and MLT on the immune-expression levels of caspase-3 (E), p53 (F), Bax (G) and Bcl-2 (H) in the pancreatic tissues of rats in the different groups.

**Figure** **S8** Share computer assisted digital image analysis (digital morphometric analysis) of histological heart sections in the different groups.

**Figure** **S9** Share computer assisted digital image analysis (digital morphometric analysis) of histological pancreatic sections islets of Langerhans blot area and β cells count within the blot area in the different groups.

**Figure** **S10** Share computer assisted digital image analysis (digital morphometric analysis) of Masson trichrome stained heart sections in the different groups.
