# Supplementary figures and images for: Melatonin protects the heart and pancreas by improving glucose homeostasis, oxidative stress, inflammation and apoptosis in T2DM-induced rats
Source: Heliyon. 2021 Mar 12;7(3):e06474. doi: 10.1016/j.heliyon.2021.e06474 (PMC7970364; doi:10.1016/j.heliyon.2021.e06474)

## Slide 1
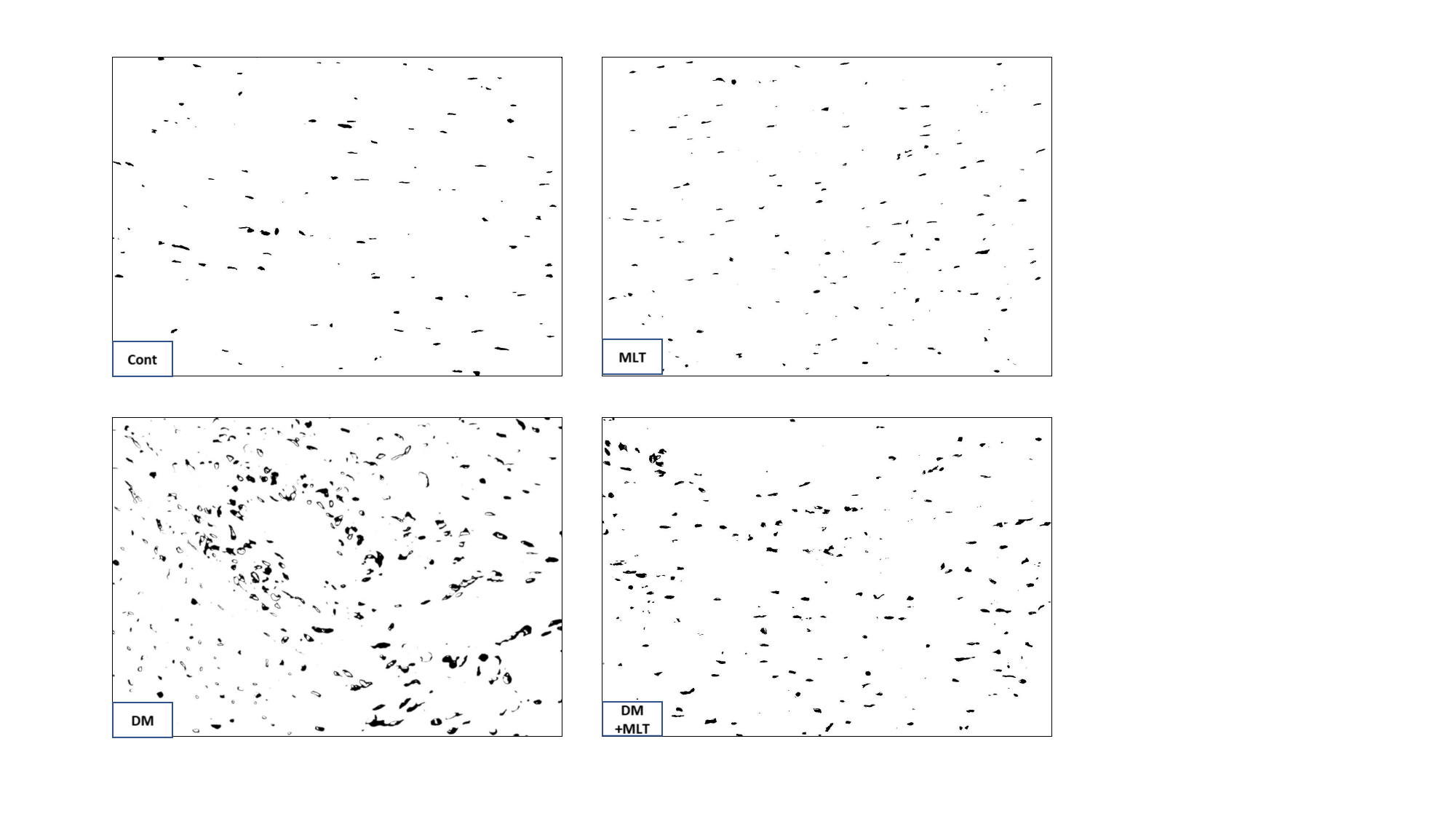

Supplement: Figure S8 [file mmc9.pptx]

## Slide 1
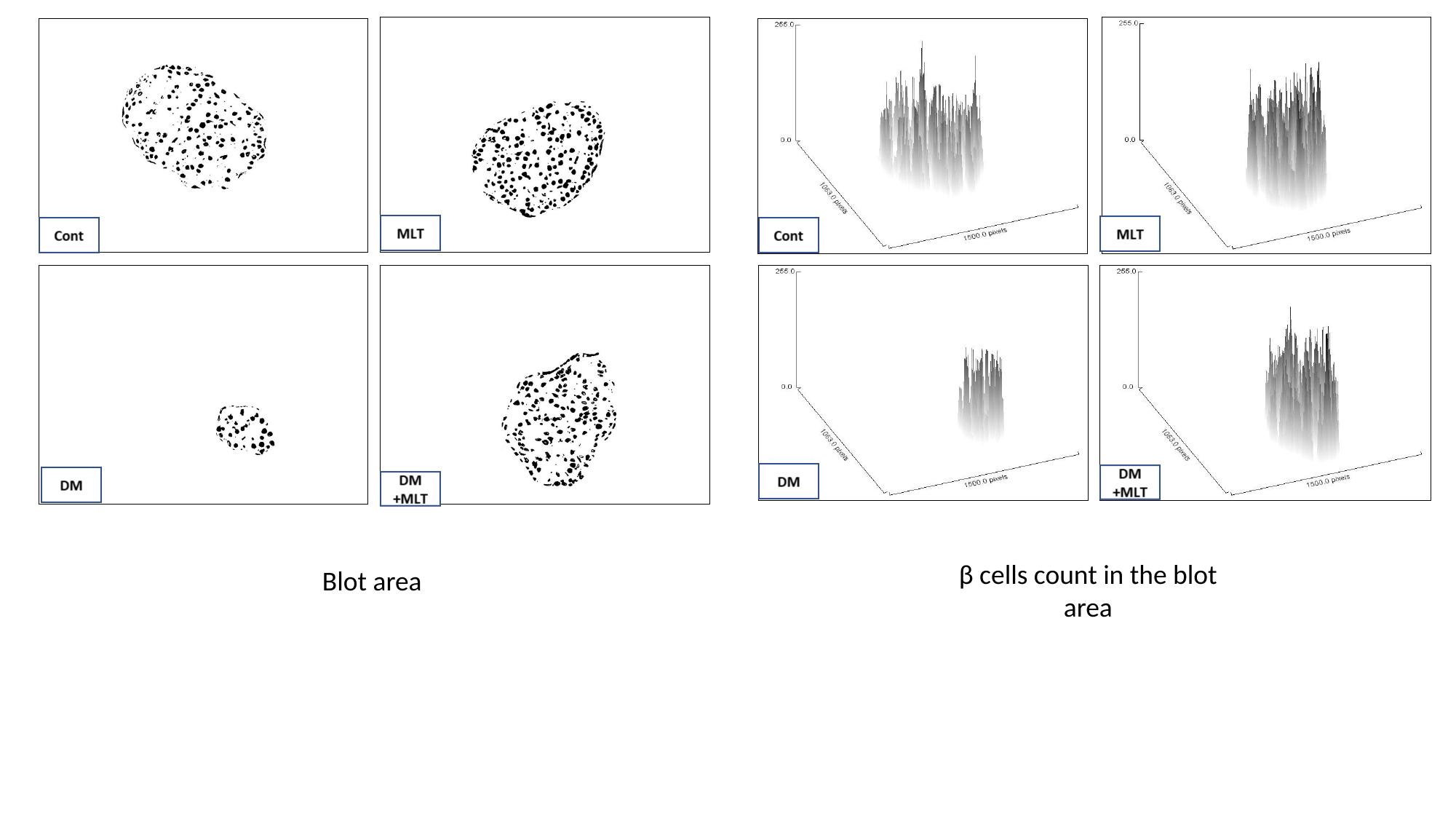

β cells count in the blot area
Blot area

Supplement: Supplementary file 10 — Figure S9 [file mmc10.pptx]

## Slide 1
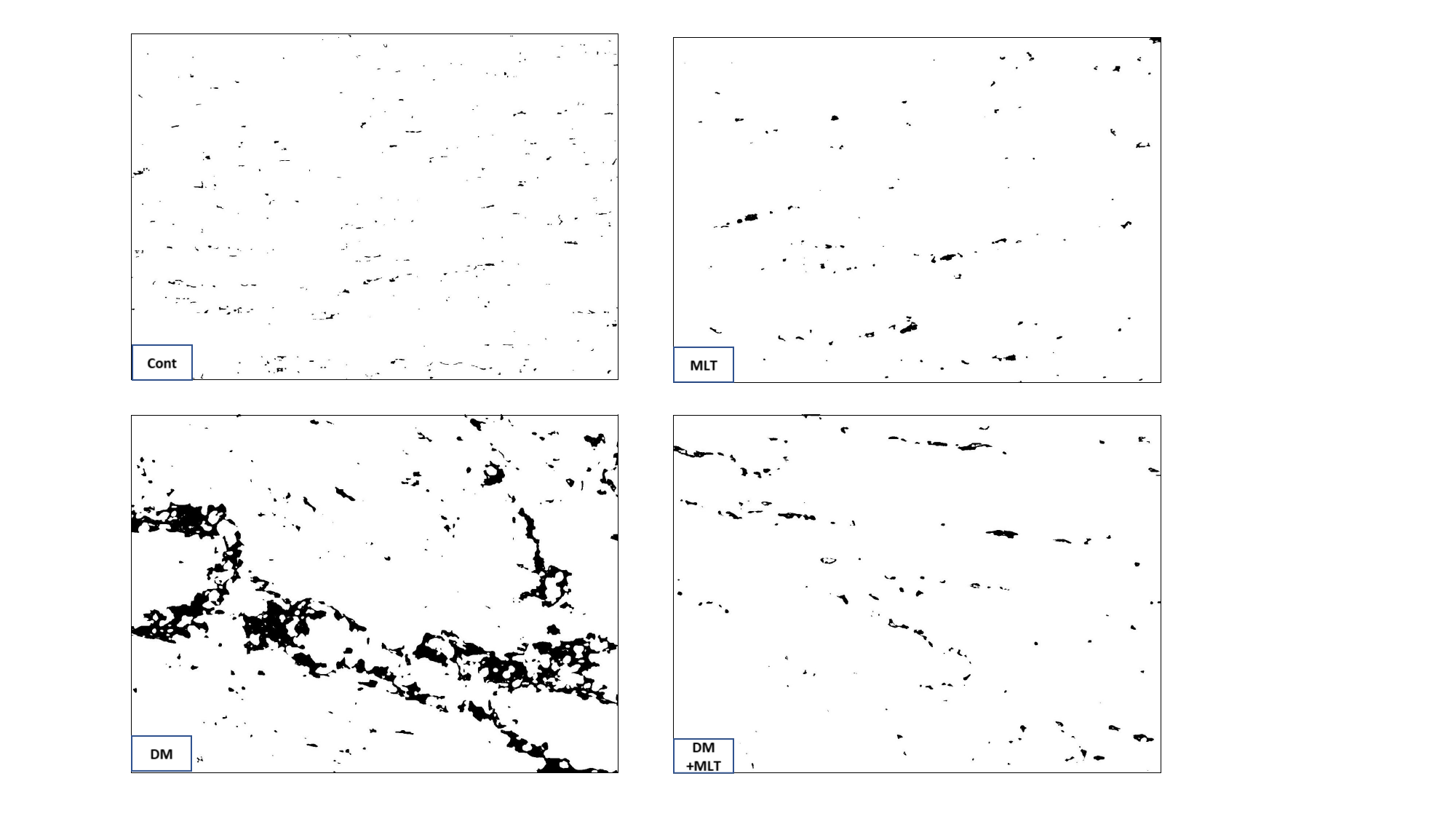

Supplement: Figure S10 [file mmc11.pptx]
